# Multiple molecular interactions redundantly contribute to RB-mediated cell cycle control

**DOI:** 10.1186/s13008-017-0029-6

**Published:** 2017-03-14

**Authors:** Michael J. Thwaites, Matthew J. Cecchini, Srikanth Talluri, Daniel T. Passos, Jasmyne Carnevale, Frederick A. Dick

**Affiliations:** 1 London Regional Cancer Program, London, Canada; 2 Children’s Health Research Institute, London, Canada; 3 Department of Biochemistry, Western University, London, ON Canada

**Keywords:** Cell cycle, Tumor suppressor, Structure–function, Retinoblastoma, Systems biology

## Abstract

**Background:**

The G1-S phase transition is critical to maintaining proliferative control and preventing carcinogenesis. The retinoblastoma tumor suppressor is a key regulator of this step in the cell cycle.

**Results:**

Here we use a structure–function approach to evaluate the contributions of multiple protein interaction surfaces on pRB towards cell cycle regulation. SAOS2 cell cycle arrest assays showed that disruption of three separate binding surfaces were necessary to inhibit pRB-mediated cell cycle control. Surprisingly, mutation of some interaction surfaces had no effect on their own. Rather, they only contributed to cell cycle arrest in the absence of other pRB dependent arrest functions. Specifically, our data shows that pRB–E2F interactions are competitive with pRB–CDH1 interactions, implying that interchangeable growth arrest functions underlie pRB’s ability to block proliferation. Additionally, disruption of similar cell cycle control mechanisms in genetically modified mutant mice results in ectopic DNA synthesis in the liver.

**Conclusions:**

Our work demonstrates that pRB utilizes a network of mechanisms to prevent cell cycle entry. This has important implications for the use of new CDK4/6 inhibitors that aim to activate this proliferative control network.

## Background

Uninhibited cellular division is a feature of cancer cells. As such, pathways that regulate proliferation are typically disrupted in human cancer [1]. At a molecular level, the cell division cycle is frequently controlled by decisions made in the G1 phase [2]. Once through this phase, the cell is committed to DNA replication and ultimately completion of cell division. The retinoblastoma gene product (pRB) has been shown to be a key regulator of the restriction point that is responsible for controlling S-phase entry [3]. The best known function of pRB is the repression of E2F transcription factor activity [4]. RB performs this function by directly binding the transactivation domain of E2Fs, preventing the recruitment of transcriptional activators to influence gene transcription [4]. In addition, pRB can recruit chromatin regulating enzymes, such as histone deacetylases, to assist in transcriptional repression [5]. This blocks gene expression that is necessary for DNA synthesis and cell cycle entry [2]. In the presence of mitogens cyclin dependent kinases phosphorylate pRB, changing its conformation and releasing E2Fs [6]. Free E2Fs are then able to stimulate transcription and S-phase progression. While this model describes cell cycle entry quite accurately, the role for the same molecular interactions between pRB and E2Fs in cell cycle exit is less clear as pRB dependent arrest can occur much faster than E2F repression [7].

The minimal interaction domain that mediates stable E2F binding to pRB is the large pocket, and this fragment is also the minimal growth suppressing domain [8, 9]. The large pocket is composed of three regions called A, B, and C [3]. The A and B domains of pRB form the pocket in which the transactivation domain of E2Fs bind [10, 11]. In addition, pRB interacts with a number of chromatin regulators, including HDAC containing complexes, through a well conserved interaction site on the B box of pRB known as the LxCxE binding cleft [5]. This binding site is well defined for its ability to contact the LxCxE motif in viral oncoproteins [12]. Simultaneous interactions between E2Fs, pRB, and chromatin regulators through LxCxE interactions form the basis of active transcriptional repression through E2Fs. The C-terminus of pRB is largely unstructured and serves as a contact point for numerous protein interactions [3, 13]. It is required for stable interaction with E2F-DP dimers [14], as well as a unique interaction with the marked box domain of E2F1 [15]. Analysis of the large pocket of pRB has contributed to our knowledge of E2Fs in cell cycle control. However, there is little to reconcile how multiple competing protein interactions through this domain contribute to pRB’s overall influence on cell proliferation.

Genetic ablation of RB causes defects in cell proliferation control in tissues and in primary cell culture experiments [16, 17]. However, early studies of pRB-mediated cell cycle regulation exploited the RB null SAOS2 osteosarcoma cell line [8, 9, 18]. RB expression in these cells leads to a robust accumulation of 2 N DNA content, indicating a G1 arrest [19]. These studies looked at a variety of mutant versions of pRB in which strong cancer derived mutations were functionless, but low penetrance RB mutations retained the ability to at least partially restrict cell cycle entry [8, 9, 20, 21]. Surprisingly, the low penetrance mutation R661W was defective for E2F binding, but retained the ability to inhibit cell cycle entry [20–22]. More recently, a number of studies have shown that the R661W mutant can regulate cyclin dependent kinase activity through p27, independent of E2F transcriptional control [7, 23]. Importantly, these studies established that the LxCxE binding cleft and C domains within the large pocket also mediate interactions with the anaphase promoting complex and Skp2 to stabilize p27 expression [7, 24]. Surprisingly, a unified model of how E2F dependent and independent proliferative control mechanisms interact has yet to emerge.

To understand the importance of different protein interaction points in the RB large pocket, targeted mutations to disrupt the LxCxE binding cleft [25–28], the canonical E2F binding site [29, 30], and pRB’s unique interaction with E2F1 in the C-terminus [31, 32], have been generated in mice. Analysis of proliferation in cells and tissues from these mutant animals suggests that individual protein interactions play context specific roles. For example, LxCxE binding cleft mutant mice (called *Rb1* ^*L*^, or *Rb1* ^*NF*^) are viable with hyper proliferation largely limited to mammary ductal epithelium, that is likely due to unresponsiveness to growth inhibitory signals from TGF-β [33]. Importantly, these mice are not spontaneously cancer prone [27, 34], and they are capable of blocking E2F transcription under a number of physiological circumstances [35]. However, repression of E2F targets is diminished following DNA damage, and the ability of these cells to enter senescence is compromised [35, 36]. Furthermore, mutagen treatment induces cancer in these mice under conditions where E2F repression fails [26]. Disruption of pRB’s unique E2F1 interaction in mice (called *Rb1* ^*S*^) shows no detectable change in proliferative control in tissues or isolated cells [32]. Lastly, mutational disruption of pRB–E2F interactions in *Rb1* ^*G/G*^ mice results in cells with accelerated entry into the cell cycle, but normal cell cycle exit [29, 30]. Remarkably, this mutation does not predispose mice to cancer [29], however, disruption of this interaction in combination with p27 deficiency deregulates cell cycle arrest functions and these mice are highly cancer prone [30]. This result is also provocative because the cell cycle arrest defects in *Rb1* ^*G/G*^; p27 deficient compound mutants aren’t found in either single mutant strain alone. These data suggest that pRB dependent cell cycle arrest may depend on a complex network of proliferative control signals such that loss of individual functions have limited effect on their own. This concept is underscored by the fact that no targeted knock in strain recapitulates the complete proliferative control and cancer susceptibility phenotypes of *Rb1* ^−*/*−^ mice. In this manuscript, we aimed to eliminate individual binding surfaces in the pRB large pocket to determine the extent that each contributes to cell cycle control alone and in combinations using SAOS2 arrest as a read out. Here, we demonstrate that multiple individual binding surfaces in the large pocket contribute to pRB-mediated cell cycle control in cell culture, and provide proof of principle that this network functions endogenously to regulate DNA replication in the liver.

## Methods

### GST pulldowns and western blotting

C33A cells were transfected with either HA-E2F1-3 (along with DP1), myc tagged CDH1 or pRB expression plasmids under the control of CMV promoters using standard calcium phosphate precipitation techniques. 40 h after transfection cells were washed and collected in GSE buffer (20 mM Tris, pH 7.5, 420 mM NaCl, 1.5 mM MgCl_2_, 0.2 mM EDTA, 25% glycerol, 5 µg/mL leupeptin, 5 µg/mL aprotinin, 0.1 mM Na_3_VO_4_, 0.5 mM NaF, and 1 mM DTT) and frozen at −80 °C. Cell extracts were centrifuged and the supernatant was diluted twofold in low salt GSE (20 mM Tris, pH 7.5, 1.5 mM MgCl_2_, 0.2 mM EDTA, 25 mM DTT, 0.1% NP-40) and combined with glutathione beads and recombinant fusion proteins. GST-RB large pocket (amino acids 379–928) and GST-HPV-E7 recombinant proteins were expressed and purified as previously described [29]. Beads were then washed twice with low salt GSE, boiled in SDS-sample buffer, resolved by SDS-PAGE, and western blotted. HA-tagged proteins were detected using anti-HA 3F10 (Roche), myc-tagged CDH1 was detected using monoclonal antibody 9E11, and pRB was detected with G3-245 (BD Pharmagen). In order to test pRB stability, cells transfected with CMV expressed pRB were treated with 100 µg/mL cycloheximide for 24 h. Extracts were prepared in GSE buffer every 3 h up to 15 h. Extracts were spun down and western blotted for pRB.

### SAOS2 cell cycle arrest assays

SAOS2 cells were transfected and harvested as previously described [37]. Briefly 10^6^ cells were plated in 6 cm dishes and transfected with 0.15 µg of CMV-pRB, 1 µg of CMV-CD20 and 3.85 µg of CMV-β-gal, or 1 µg of CMV-CD20 and 4 µg of CMV-β-gal as a negative control, using X-tremeGENE transfection reagent (Roche). Cells were re-plated onto 10 cm dishes 24 h after transfection, and harvested 48 h later. Cells were then stained with a fluorescein conjugated anti-CD20 antibody to mark successfully transfected cells, as well as with propidium iodide (PI) to determine their DNA content. Flow cytometry was then performed to identify the percentage of CD20 positive cells with 2 N DNA content as a measure of G1. In experiments expressing cell cycle arrest as percent change in G1, arrest data was scaled using CMV-pRB and CMV-β-gal as standards for maximal increase and unchanged G1 content allowing comparisons between different batches of experiments.

### Animal housing, dissection and histology

All animals were housed and handled as approved by the Canadian Council on Animal Care. Mice were sacrificed at 8 weeks of age, dissected, and livers were processed for downstream applications. For histology, livers were fixed in formalin for 72 h followed by 72 h in PBS before being stored in 70% ethanol. Livers were then embedded in paraffin and five micron sections were cut and stained with Hematoxylin and Eosin. Images were captured on a Zeiss Axioskop 40 microscope and Spot Flex camera, and nuclear area in the livers was calculated using EyeImage software (Empix Imaging, Mississauga, Ontario, Canada).

### Ploidy analysis of adult livers

A small piece of frozen liver was added to buffer A (25 mM Tris pH 7.5, 50 mM KCl, 2 mM MgCl_2_, 1 mM EDTA, 1 mM PMSF). Tissue was ground on ice with a mechanical tissue grinder. Tissue was then homogenized using a 1 mL dounce homogenizer and tight pestle. Nuclei were centrifuged at 12,000×*g*, then washed in buffer A and centrifuged. The pellet was then resuspended in Propidium Iodide solution (0.5 mg/mL PI, 0.1% NP-40, 0.1% sodium citrate, 40 µg/mL RNase A in PBS). Samples were then analyzed by flow cytometry using standard methods to quantitate DNA content.

### RNA isolation and E2F target gene quantification

RNA from livers was isolated using an RNeasy fibrous tissue kit (Invitrogen). Expression levels of the E2F target genes, *Pcna, Ccne1* (cyclin E1)*, Ccna2* (cyclin A2)*, Tyms* (thymidylate synthase)*, Mcm3*, and *Rbl1* (p107), were determined using the Quantigene Plex 2.0 reagent system from Affymetrix (Santa Clara, CA) and a BioPlex200 multiplex analysis system as previously reported [38]. Expression levels were normalized to the expression of β-actin.

### BrdU staining of tissue sections

To analyze DNA replication, mice were injected with 200 μL of 16 µg/mL BrdU (Sigma) in their peritoneal cavity 2 h before sacrifice. Livers were then isolated, fixed in formalin, embedded, and sectioned according as above. Sections were deparaffinized and rehydrated using a series of xylene and ethanol washes. The sections were brought to a boil in sodium citrate buffer and then maintained at 95 °C for 10 min. The cooled sections were rinsed in water three times for 5 min, and then rinsed in PBS for 5 min. The sections were blocked in phosphate buffered saline (PBS) supplemented with 2.5% horse serum and 0.3% Triton X-100 for 1 h. The sections were then incubated with anti-BrdU antibodies (BD-Biosciences) in blocking buffer overnight at 4 °C and rinsed in PBS three times for 5 min each time. The slides were incubated with horse anti-mouse immunoglobulin G-fluorescein isothiocyanate (Vector) for 1 h and rinsed in PBS. The slides were then mounted with Vectashield plus DAPI (Vector). Fluorescent images were captured on a Zeiss Axioskop40 microscope and Spot Flex camera and colored using EyeImage software (Empix Imaging, Mississauga, Ontario, Canada), or a similar system.

## Results

### A cell culture assay demonstrates molecular redundancy of RB functions in proliferative control

**Fig. 1:**
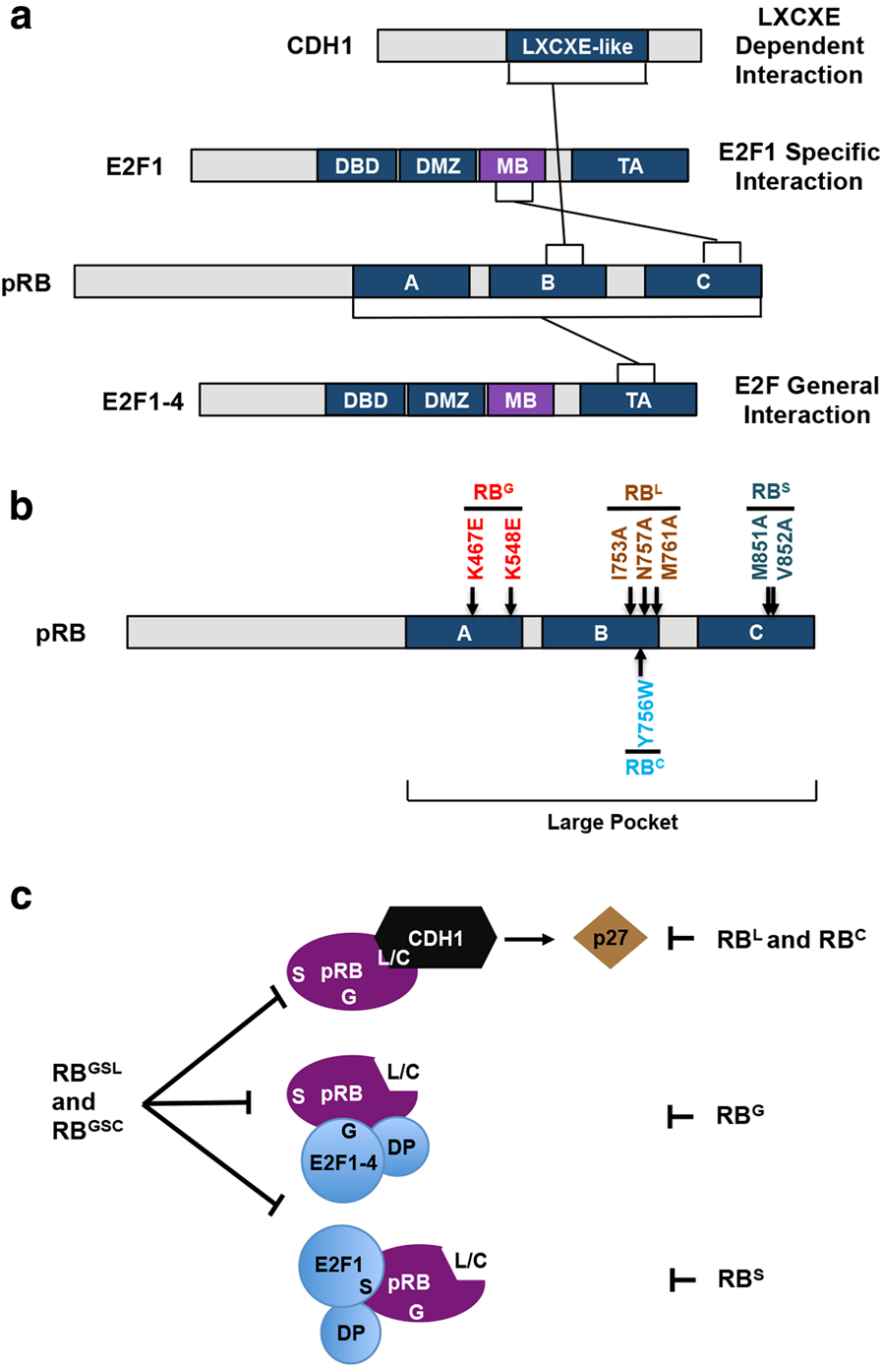
Interaction domains located in the large pocket of pRB and substitutions used in this study. **a** Linear diagrams of open reading frames for the indicated proteins highlighting the regions that mediate interactions with pRB. pRB can bind E2F1-4 through the transactivation domain in the C-terminus of E2Fs known as the ‘general’ interaction. Alternatively, pRB can also bind the marked box domain of E2F1 through its C-terminal domain, termed the RB-E2F1 ‘specific’ interaction. **b** Locations of point mutations within the pRB open reading frame used in this study. RB^G^ refers to mutations that disrupt the E2F general interaction, RB^S^ is a mutation that disrupts the E2F1 specific interaction. RB^C^ and RB^L^ both disrupt interactions through the LxCxE binding cleft. All codon numbers correspond to the human sequence. The large pocket domain is amino acids 379–928. **c** Diagram depicting the cell cycle control mechanisms that can be influenced by the 3 pRB binding surface mutations used in this study

Tumor suppression by the retinoblastoma protein has typically been associated with its ability to block cell cycle progression and repress E2F transcription factors [4]. However, defective E2F binding by pRB has been shown to have modest effects on proliferative control in SAOS2 cell culture experiments [15, 20–22], and gene targeted mouse models [29, 30]. In an attempt to describe the molecular interactions necessary for pRB-mediated cell cycle arrest we investigated forms of pRB that were individually mutated at each of three distinct binding surfaces in the large pocket; the general E2F binding site (RB^G^), the E2F1 specific site (RB^S^), and the LxCxE binding cleft (using either the RB^L^ or RB^C^ mutations). Figure 1a diagrams pRB protein interactions and shows the relevant regions in each open reading frame that participate. Amino acid substitutions that are demonstrated to disrupt these contacts are shown in Fig. 1b [24, 37, 39, 40], along with single letter nomenclature for each allele (e.g. RB^G^). Lastly, the types of interactions between pRB and E2Fs, or LxCxE motif proteins, are illustrated with the alleles that disrupt them individually shown on the right, and the intended effect of a combined mutant allele on the left (Fig. 1c).

**Fig. 2:**
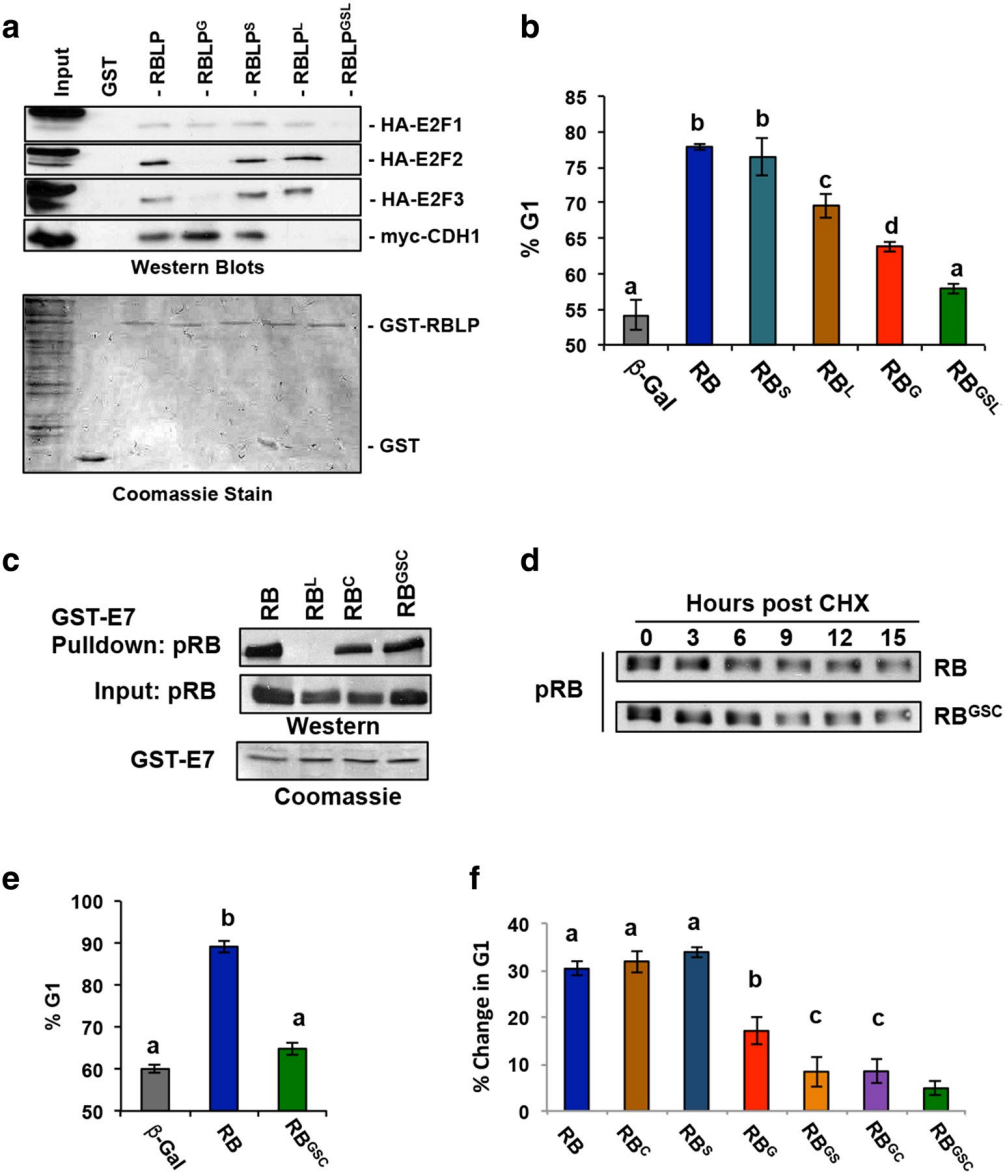
Multiple point mutations are needed to overcome RB mediated cell cycle arrest. **a** GST-tagged RB large pocket proteins corresponding to the RB^G^, RB^S^, RB^L^, and RB^GSL^ mutant versions of pRB were produced and purified. These GST-fusions were incubated with C33A extracts transfected with the indicated expression constructs. Bound proteins were isolated by precipitation and identified by western blotting. **b** Constructs containing full-length RB harboring the indicated mutations under the control of a CMV promoter were transfected into SAOS2 cells along with a CD20 reporter. Cells were then stained with propidium iodide and the percentage of cells in G1 were determined by DNA content of CD20 positive cells. *Bars* indicate the mean of three separate experiments, and error bars indicate one standard deviation from the mean. *Letters* indicate groups that are significantly different from one another (ANOVA, Tukey test, p < 0.05). **c** Full length CMV-RB constructs were transfected into C33A cells and extracts were incubated with recombinant HPV-E7. Bound proteins were isolated by precipitation and western blotted to detect pRB. **d** Full length RB^WT^ and RB^GSC^ were transfected into C33A cells prior to cycloheximide treatment (CHX). Extracts were prepared over a 15 h time course and stability was monitored by Western blotting. **e** Constructs containing full-length RB harboring the various mutations, or combinations of mutations, under the control of a CMV promoter were transfected into SAOS2 cells along with a CD20 reporter. Cells were then stained with propidium iodide and the percentage of cells in G1 were determined by DNA content of CD20 positive cells. *Bars* indicate the mean of three separate experiments, and error bars indicate one standard deviation from the mean. **f** Transfections and cell cycle analysis were performed as in **b** and **e**, except the increase in G1 cells is shown as Change in % G1 (relative to β-Gal control). *Letters* indicate groups that are significantly different from one another (ANOVA, Tukey test, p < 0.05)

GST-tagged versions of the pRB large pocket (GST-RBLP, pRB amino acids 379–928) containing the 3 mutations described above, as well as the triple mutant, were produced in bacteria. GST pulldowns were performed to test interaction defects predicted to occur in these mutants (Fig. 2a). RB deficient C33A lysates derived from transfections with the indicated E2Fs, or CDH1 were produced and used in pulldown experiments. As expected the RB^G^ mutation disrupts binding of the activator E2Fs, E2F2 and E2F3. RB^L^ disrupts the LxCxE binding cleft and is defective for binding the anaphase promoting complex targeting subunit CDH1. Finally, since E2F1 is capable of associating with pRB through two qualitatively different interactions, the general site and the specific site, binding is only lost following mutation of both sites in the triple mutant RB^GSL^. Full length pRB constructs containing these mutations were then transfected into SAOS2 cells to determine their effectiveness in causing a G1 cell cycle accumulation. As previously shown, expression of wild-type pRB in SAOS2 cells lead to a build up of cells in G1 as determined by propidium iodide staining and flow cytometry (Fig. 2b) [19]. Expression of the mutant constructs of pRB had various levels of effectiveness for inducing a G1 cell cycle arrest (Fig. 2b). Notably, the RB^S^ mutation showed a similar ability to block proliferation as wild-type RB (Fig. 2b). By contrast, disruption of the general binding pocket in the RB^G^ mutant, or disruption of the LxCxE binding cleft (RB^L^) resulted in a significant, but partial decrease in the percentage of cells in the G1 phase of the cell cycle (Fig. 2b). Importantly, no individual mutation is able to completely disrupt RB function. However, when all three mutations were combined into one pRB molecule (RB^GSL^), the ability of pRB^GSL^ to induce a G1 arrest was not statistically different from that of the β-Gal negative control (Fig. 2b). As disruption of the various interactions lead to an inability of pRB to bind to any of its LxCxE or E2F interactors, we next aimed to confirm that combination mutations led to disruption of these binding surfaces, as opposed to simply disrupting pRB structure or stability. To address this possibility, we used the RB^C^ mutation that retains the ability to associate with HPV-E7, but has previously been shown to be defective for its interaction with CDH1 [24]. Figure 2c demonstrates that both the RB^C^, and an RB^GSC^ combination were able to maintain RB-E7 interaction, suggesting this mutant combination retains it structure. Furthermore, the stability of the RB^GSC^ mutation was determined by expressing both RB^WT^ and RB^GSC^ in C33A cells. Cells were then treated with cycloheximide and protein was isolated over a period of 15 h. Western blots confirmed that RB^WT^ and RB^GSC^ have equal stability, further suggesting that these substitutions do not result in the misfolding and hence pleiotropic loss of pRB function (Fig. 2d). Finally, SAOS2 cell cycle arrest assays were performed using the RB^C^ mutant alone or in double and triple combinations (RB^GC^ or RB^GSC^). As with the RB^GSL^ mutant, the triple mutant combination RB^GSC^ was unable to increase the proportion of G1 cells beyond that of β-Gal controls (Fig. 2e). In addition, SAOS2 cell cycle arrest following transfection with the RB^GC^ and RB^GS^ double mutants diminished the ability to induce a G1 cell cycle arrest beyond any single mutant, but was less detrimental than the RB^GSC^ combination (Fig. 2f). These results demonstrate that pRB’s activity in this arrest assay can be defined through loss of individual protein interactions.

**Fig. 3:**
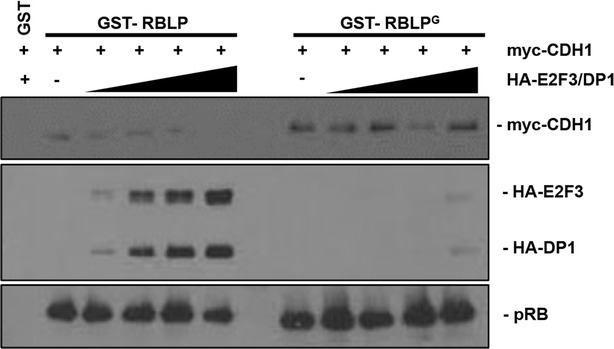
Competition between E2Fs and CDH1 for pRB binding. Purified GST-RBLP or an -RBLP^G^ mutant was incubated with constant levels of myc-CDH1, and increasing quantities of HA-tagged E2F3/DP1 from transfected lysates. GST-pulldowns were performed and associated levels of myc-CDH1 and HA-E2F3/DP1 were determined by western blotting. Western blots using anti-pRB antibodies show the levels of GST-RBLP proteins precipitated in each experiment

The combination of RB^G^ and RB^L^ mutations in RB^GSL^ is more severe than either alone (Fig. 2b). It is difficult to envision LxCxE interaction defects enhancing loss of function of pRB-E2F binding defects through transcriptional control since the RB^G^ mutation already disrupts recruitment to E2F promoters [29, 30]. For this reason, we investigated non-E2F dependent mechanisms that could be lost because of the RB^L^ mutation such as binding to CDH1. In order to investigate how E2F and CDH1 dependent arrest mechanisms may relate to one another, we tested pRB’s ability to interact with each simultaneously. For this experiment we mixed C33A extracts containing myc-tagged CDH1 with increasing amounts of HA-E2F3/DP1 extracts and tested their ability to bind to GST-RBLP in pulldown experiments (Fig. 3). This experiment reveals that increasing quantities of HA-E2F3/DP1 prevent myc-CDH1 from binding to GST-RBLP (Fig. 3, left side). Disruption of E2F3 binding to pRB using a GST-RBLP^G^ mutant prevents competition with myc-CDH1 for binding to pRB. This experiment suggests that pRB is unable to engage E2F3 and CDH1 dependent functions simultaneously, suggesting that these functions are interchangeable. This mirrors findings from recent in vivo approaches to pRB dependent cell cycle control [30], and this will be explored further in the discussion.

### A compound mutant mouse model demonstrates molecular redundancy in RB control of DNA replication

Mutation of all three binding surfaces in the RB large pocket was required to maximally impair RB mediated cell cycle control (Fig. 2b, e). This finding, combined with the fact that individual mutations for each of these binding sites in gene targeted mice did not phenocopy the *Rb1* ^−*/*−^ proliferative control defects in primary cell culture, suggests that the function of pRB in cell cycle control may be composed of several distinct mechanisms [28, 29, 32]. To approximate the dysfunction of the RB^GSL^ mutation in vivo as diagrammed in Fig. 1c, we combined our previously published *Rb1* ^*G/G*^ animals that disrupts RB-E2F interactions with p27 null mice (*Cdkn1b* ^−*/*−^) to eliminate its influence on cell cycle control [30]. In addition, we crossed these mice into an E2F1 null background to eliminate any effect on cell cycle regulation by the pRB-E2F1 specific interaction. This combination of mutations *Rb1* ^*G/G*^ *; Cdkn1b* ^−*/*−^ *; E2f1* ^−*/*−^, represents one potential scenario of the effects of the RB^GSL^ mutation in vivo on cell cycle control. Interestingly, *Rb1* ^*G/G*^ *; Cdkn1b* ^−*/*−^ *; E2f1* ^−*/*−^ (triple mutant) animals are viable and occur at normal Mendelian ratios (Table 1).

**Table 1:** Frequency of compound mutant mice

*E2f1* ^−*/*−^; *Rb1* ^*G/*+^ *; Cdkn1b* ^+*/*−^ × *E2f1* ^−*/*−^; *Rb1* ^*G/*+^ *; Cdkn1b* ^+*/*−^	
Genotype	P14
*E2f1* ^−*/*−^ *; Rb1* ^+*/*+^ *; Cdkn1b* ^+*/*+^	8 (13)
*E2f1* ^−*/*−^ *; Rb1* ^+*/*+^ *; Cdkn1b* ^+*/*−^	29 (26)
*E2f1* ^−*/*−^ *; Rb1* ^+*/*+^ *; Cdkn1b* ^−*/*−^	12 (13)
*E2f1* ^−*/*−^ *; Rb1* ^*G/*+^ *; Cdkn1b* ^+*/*+^	29 (26)
*E2f1* ^−*/*−^ *; Rb1* ^*G/*+^ *; Cdkn1b* ^+*/*−^	64 (52)
*E2f1* ^−*/*−^ *; Rb1* ^*G/*+^ *; Cdkn1b* ^−*/*−^	9^a^ (26)
*E2f1* ^−*/*−^ *; Rb1* ^*G/G*^ *; Cdkn1b* ^+*/*+^	12 (13)
*E2f1* ^−*/*−^ *; Rb1* ^*G/G*^ *; Cdkn1b* ^+*/*−^	35 (26)
*E2f1* ^−*/*−^ *; Rb1* ^*G/G*^ *; Cdkn1b* ^−*/*−^	10 (13)
Total	208

The indicated genotypes of mice were crossed and all resulting progeny were genotyped. The number of live animals obtained at 2 weeks of age is indicated for each genotype and the expected number based on Mendelian inheritance is indicated in brackets

^a^ Indicates significance as determined by Chi squared test

**Fig. 4:**
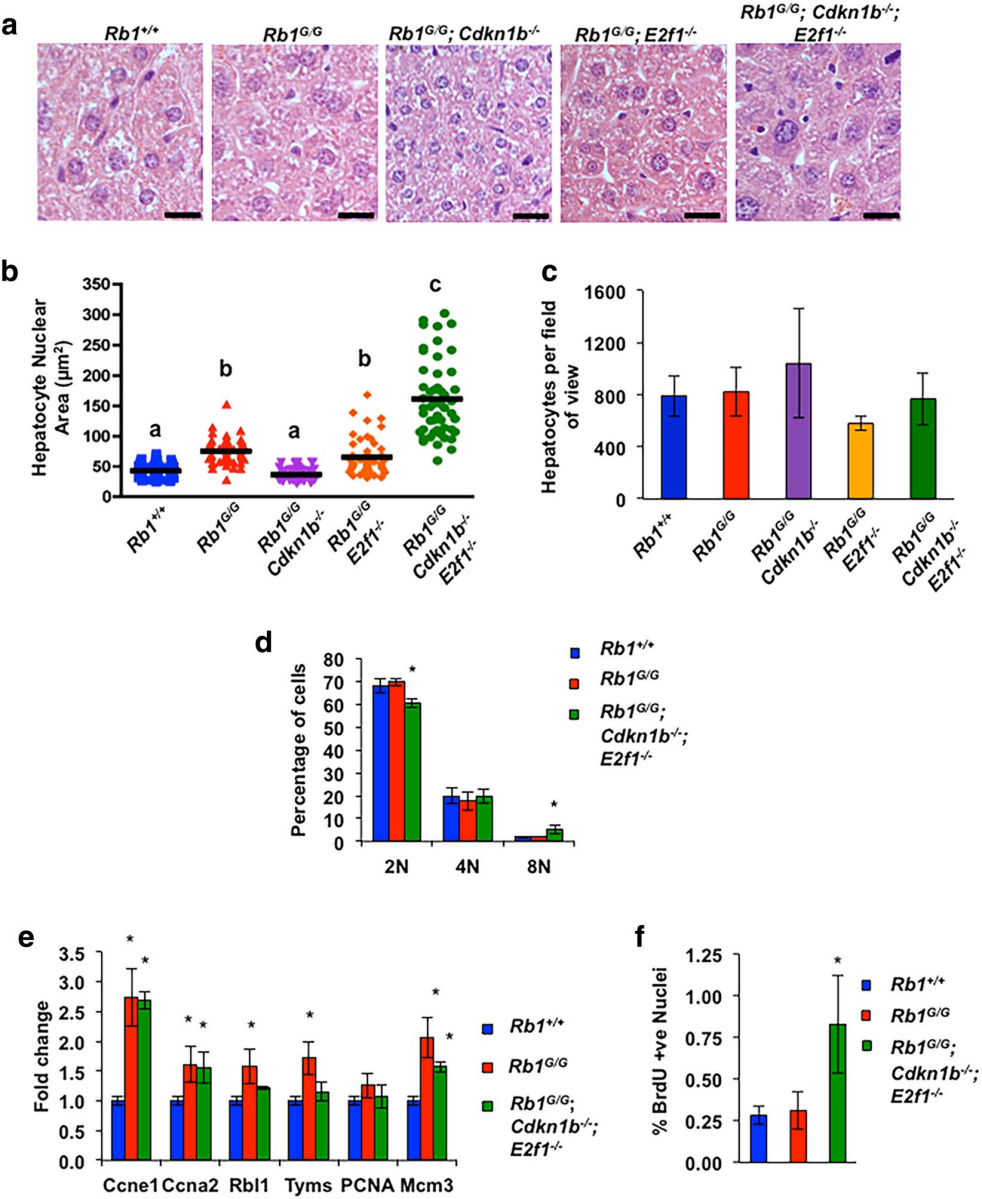
Ectopic DNA-replication in *Rb1* ^*G/G*^ *; Cdkn1b* ^−*/*−^ *; E2f1* ^−*/*−^ compound mutant mice. **a** H&E staining of liver sections from 8-week old wild type, *Rb1* ^*G/G*^, double mutant *Rb1* ^*G/G*^ *; Cdkn1b* ^−*/*−^, and *Rb1* ^*G/G*^ *; E2f1* ^−*/*−^ mice, as well as *Rb1* ^*G/G*^ *; Cdkn1b* ^−*/*−^ *; E2f1* ^−*/*−^ triple mutant animals. The *scale bars* represent 20 μm. **b** Nuclear size from the images in **a** was determined and the mean size is indicated. Measurements were made from at least 50 nuclei, *a*, *b*, *c* represents statistically different groups as determined by ANOVA followed by Tukey’s test (p < 0.05). **c** Total number of hepatocytes per 20X field of view was averaged from the indicated genotypes. No statistical differences were observed by AVONA followed by Tukey’s test (p < 0.05) **d** Nuclei were extracted from livers, stained with propidium iodide, and analyzed for DNA content by flow cytometry. **e** The relative expression level of six E2F cell cycle target genes from wild type, *Rb1* ^*G/G*^, and *Rb1* ^*G/G*^ *; Cdkn1b* ^−*/*−^ *; E2f1* ^−*/*−^ was determined from RNA extracted from 8-week-old livers. **f** 8-week-old mice were pulse labeled with BrdU 2 h prior to sacrifice and livers were sectioned and stained for BrdU. The percentage of BrdU positive nuclei was determined. At least 500 nuclei were counted per mouse. All bar graphs represent at least 3 individual experiments, and *error bars* indicate one standard deviation from the mean. An *asterisk* represents a statistically significant difference from the wild type control (*t* test, *P* < 0.05)

Since triple mutant mice did not phenocopy the embryonic lethality seen in *Rb1* ^−*/*−^ animals we next sought to determine if any tissues display loss of cell cycle control [41]. Previously, Mayhew et al. showed that tissue specific knockout of pRB in the murine liver resulted in the up regulation of E2F target genes and ectopic DNA replication, endoreduplication, and accumulation of nuclei with elevated ploidy [42]. Since hepatocytes often endoreduplicate it is possible to detect the accumulation of misregulated DNA replication over time [35]. We therefore, aimed to analyze aspects of cell cycle control in the livers of *Rb1* ^*G/G*^ *; Cdkn1b* ^−*/*−^ *; E2f1* ^−*/*−^ animals to determine if these mutations were capable of disrupting pRB control of DNA replication. H&E staining of livers revealed that hepatocyte triple mutant adult livers had enlarged nuclei that on average were three times larger that wild-type and *Rb1* ^*G/G*^ *; Cdkn1b* ^−*/*−^ double mutant animals as well as twice as large as *Rb1* ^*G*/G^ and *Rb1* ^*G/G*^ *; E2f1* ^−*/*−^ nuclei (Fig. 4a, b). We also quantitated the density of hepatocytes per microscopic field of view and did not see significant differences between genotypes (Fig. 3c). Since nuclear area in liver histology correlates with DNA content [43], this suggested elevated levels of endoreduplication in *Rb1* ^*G/G*^ *; Cdkn1b* ^−*/*−^ *; E2f1* ^−*/*−^ triple mutant livers. To test whether our triple mutant had elevated ploidy in their hepatocytes, nuclei were extracted from livers of *Rb1* ^+*/*+^ *, Rb1* ^*G/G*^, and *Rb1* ^*G/G*^ *; Cdkn1b* ^−*/*−^ *; E2f1* ^−*/*−^ mice, stained with propidium iodine, and analyzed by flow cytometry for DNA content. Consistent with previous results we found that *Rb1* ^+*/*+^ livers at 8 weeks of age display very low levels of 8 N DNA content, however triple mutant livers displayed a significant increase in the level of 8 N DNA at this time point (Fig. 4c), that is similar to what is reported when *Rb1* is conditionally deleted in this organ [42]. This increase in nuclear size and subsequent DNA content indicates that triple mutant livers undergo endoreduplication. While this is a normal phenotype for liver cells over time, this suggests that the loss of these three regulatory elements controlled by pRB results in earlier endoreduplication, potentially due to a loss of cell cycle control.

We next wanted to determine the effect of our combined mutations on the regulation of pRB functions related to cell cycle control. To accomplish this, RNA was isolated from adult livers to analyze the expression of E2F target gene transcription. Consistent with our previous findings, E2F target gene expression in *Rb1* ^*G/G*^ animals is higher than wild-type levels [29]. Interestingly, triple mutant livers show high expression of some of these target genes (Fig. 4e). However, in some cases, E2F target gene expression is unchanged from wild type and this will be discussed later. To directly measure proliferation in livers, 8-week-old animals were injected with BrdU to label nuclei with actively replicating DNA. Livers were dissected, sectioned and stained for BrdU incorporation. This analysis showed that while both *Rb1* ^*G/G*^ and *Rb1* ^*G/G*^ *; Cdkn1b* ^−*/*−^ *; E2f1* ^−*/*−^ livers display increases in the expression of E2F target genes only triple mutant livers displayed increased BrdU incorporation (Fig. 4e, f). Taken together with the increased nuclear area and 8 N DNA content in triple mutant livers, these results suggest that by mutating the general binding site of pRB, and eliminating both p27 and E2F1, we have recapitulated the DNA replication defects associated with conditional deletion of *Rb1* in adult livers. These in vivo results also mirror the effects seen in the SAOS2 arrest assays that suggest that no individual protein interaction with pRB accounts for its activity in cell cycle control. Instead, these data indicate that pRB likely sits in the center of a network of regulators that control DNA replication and cell division.

## Discussion

In this manuscript we aimed to further the understanding of pRB-mediated cell cycle control by disrupting pRB-binding interactions in the large pocket to quantitatively account for its arrest mechanisms. This structure–function analysis demonstrated that in order to disrupt the vast majority of pRB’s cell cycle arrest activity, three different binding surfaces needed to be altered. Surprisingly, no single interaction site was indispensible and disruption of some interaction sites had little effect on their own. We used a genetic cross to cripple these three aspects of pRB function endogenously and the combination caused ectopic DNA replication in the liver. This suggests that pRB may interchangeably use different protein interactions to influence cell cycle advancement. Insights and caveats of our study are discussed below.

It is difficult to predict the proliferative control defects of an *Rb1* deficient mouse beyond neonatal lethality due to muscle atrophy [44]. Interestingly, chimeric mice composed of a mixture of wild type and *Rb1* ^−*/*−^ cells are viable and demonstrate normal tissue cellularity, even in organs where *Rb1* ^−*/*−^ cells contribute extensively [17]. This study reveals that livers containing *Rb1* ^−*/*−^ hepatocytes display random, large nuclei, similar to our findings in triple mutant livers [17]. In addition, conditional ablation of *Rb1* in the livers of adult mice is reported to cause unscheduled DNA replication [42]. The increase in DNA copy number and BrdU incorporation was indicative of a loss of regulation of DNA synthesis [42]. In an effort to model the effects of the RB^GSL^ mutant in vivo, we combined *Rb1* ^*G/G*^ animals with p27 and E2F1 deficiency to produce triple mutant animals (*Rb1* ^*G/G*^ *; Cdkn1b* ^−*/*−^ *; E2f1* ^−*/*−^). This combination of mutations lead to a very similar DNA replication phenotype in the liver as complete *Rb1* deletion. While this is a similar phenotype as conditional deletion of *Rb1*, by no means does our study elucidate all that pRB pr E2Fs do to block the cell cycle in this or other tissues. We anticipate that viability of triple mutant mice suggests additional pRB dependent cell cycle arrest mechanisms likely remain functional in these animals. Another important consideration in our efforts to model the RB^GSL^ mutant in vivo is that deleting *Cdkn1b* and *E2f1* is not the equivalent to disrupting the binding sites on pRB that regulate them, as these interaction sites may have additional regulatory effects beyond the downstream targets we have chosen. In addition, loss of E2F1 could diminish cell proliferation even when entry into S-phase is deregulated and this could further complicate the interpretation of our analysis of triple mutant livers. Importantly, others have demonstrated that the choice between proliferation and endoreduplication in hepatocytes is determined by opposing effects of activator E2Fs (such as E2F1) and the E2F7 and E2F8 repressors [45, 46]. It is difficult to predict how the triple mutant combination used here would affect the regulation of this network of genes to cause a switch to endoreduplication. Future experiments using *Rb1* gene targeted mice carrying a combination of G, S, or L mutations in a single allele will help resolve some of these complexities.

We observed that some individual mutations contributed modestly to proliferative control alone, and more strongly when in combination with other substitutions. We suggest that this may be due in part to the competition between different cell cycle control mechanisms for access to pRB. For example, we demonstrate that E2F3 and CDH1 can compete for the opportunity to interact with pRB, and this is consistent with previous reports of E2F1 and CDH1 competing for pRB [47]. We suggest that CDH1 interactions with pRB are fundamentally different than other pRB interactors that contact the LXCXE binding site simultaneously with E2Fs [3]. Another way to consider redundancy of function through endogenous pRB is a gene targeted mouse model bearing an R654W mutation (the murine equivalent of the low penetrance human mutation R661W). This mutation not only disrupts E2F binding, it also compromised interactions at the LXCXE cleft [20], potentially illustrating the effects of multiple mutations in a single pRB molecule akin to RB^GC^ in our studies. Fibroblast cells from these mice possess many features of deregulated proliferation seen in *Rb1* ^−*/*−^ cells and this mutation is lethal during embryogenesis [48]. However, some aspects of pRB’s role in differentiation and its ability to respond to senescence inducing stimuli and resistance to tumor formation are retained [48, 49], suggesting that simultaneous deficiency by pRB for multiple interactions can reveal a more dramatic phenotype than loss of single interactions. This conclusion is further supported by deregulated cell cycle control and cancer incidence in *Rb1* ^*G/G*^ *; Cdkn1b* ^−*/*−^ mice [30], suggesting loss of multiple pRB dependent proliferative control pathways can be dramatically different than loss of a single pathway.

Consistent with multiple interactions needing to be compromised to abrogate cell cycle arrest by pRB, we also note that some mutations tested in this study, such as the M851A, V852A changes (RB^S^), have no effect on proliferative control in the SAOS2 assay on their own. We suggest that it may represent a latent proliferative control mechanism used by pRB, and there may be others. A long standing puzzle in the RB field has been the existence of proliferative control mechanisms that are mediated by the N-terminus of pRB, outside of the original growth suppressing large pocket domain [50–52]. Recent work has suggested that the N-terminus also plays a role in regulating DNA replication [52]. This may explain the phenotypic difference in proliferative control between *Rb1* ^−*/*−^ animals and that of triple mutant *Rb1* ^*G/G*^ *; Cdkn1b* ^−*/*−^ *; E2f1* ^−*/*−^ animals as the N-terminus is unaffected by our three mutations. There may also be redundancy between N-terminal and large pocket growth arrest mechanisms. Provocatively, there are also low penetrance mutations in human *RB1* that target this region of pRB; further suggesting the N-terminus contributes to pRB’s proliferative control and tumor suppressor functions [53]. We think that interchangeability of different pRB functions in proliferative control best explains our data and also encompasses additional work in the field that has previously been difficult to reconcile.

## Conclusions

RB dependent proliferative control is functionally inactivated in the vast majority of cancers, this study furthers our understanding of the importance of the various interaction surfaces of pRB and their roles in cell cycle control. In addition, CDK4/6 inhibitors have recently been developed to reactivate the RB-pathway in cancer [54–56]. Understanding the molecular interactions made by pRB and how they influence cell cycle control and tumor suppression is crucial to the proper implementation of these drugs. We expect that the mutational status of both pRB, as well as its regulation of p27 and E2Fs, will play a critical role in the effectiveness of these drugs. We suggest that patients whose tumor cells have pRB activatable p27 will benefit most from CDK4/6 inhibitors.

## Abbreviations

RB: retinoblastoma
pRB: retinoblastoma protein
CDK: cyclin dependent kinase
BrdU: bromodeoxyuridine
DAPI: 4′,6-diamidino-2-phenylindole
GST: glutathione-S-transferase
